# Targeting MAPK in Cancer 2.0

**DOI:** 10.3390/ijms23105702

**Published:** 2022-05-20

**Authors:** Elisabetta Rovida, Ignazia Tusa

**Affiliations:** Department of Experimental and Clinical Biomedical Sciences “Mario Serio”, University of Florence, 50134 Florence, Italy; ignazia.tusa@unifi.it

Mitogen-activated protein kinase (MAPK) pathways are prominently involved in the onset and progression of cancer. In particular, aberrant activation of MAPK pathways is mostly the consequence of activating mutations in RAS and RAF proteins that lead to the constitutive activation of the extracellular signal-regulated kinase 1/2 (ERK1/2) pathway. On the other hand, the relevance of c-Jun N-terminal kinases and p38 isoforms in promoting cancer is less univocal; thus, the targeting of this MAPK is not an established therapeutic approach. Interestingly, increasing evidence points to ERK5 as an additional possible target for cancer therapy, whereas the possibility to exploit ERK7/ERK8-targeted therapy still lacks a rationale. 

An interesting review in the “Targeting MAPK in Cancer 2.0” Special Issue addressed the role of MAPK pathways in modulating drug sensitivity and resistance in cancer, spanning from recent evidence in the ERK1/2 pathway to the mechanisms dealing with the impact of p38 and JNK MAPK on the response of cancer cells to chemotherapies and targeted therapies [1]. In this respect, starting from the fact that activation of PI3K has been described among the resistance mechanisms to RAF-MEK1/2- targeting and co-occurrence of RAF-MEK1/2 and PI3K/PTEN mutations is possible, the research article by Rittler and colleagues compared the effects of horizontal combination of the MEK inhibitor selumetinib with the PI3K/mTOR dual inhibitor BEZ235 in BRAF-only mutant and in BRAF + PI3K/PTEN double mutant cancer cells using cell lines from different types of cancers (i.e., melanoma, lung and colon adenocarcinoma), as well as spheroids and an in vivo model [2]. An updated analysis of the preclinical rationale for combining targeting of the MAPK pathway and of autophagy, as well as of the most recent clinical trials that have been launched to capitalize on this potentially synthetic lethal approach to cancer therapy, has been performed in the review from Lee and colleagues [3]. Finally, the interplay between the MAPK/ERK and the Hippo/MST pathways, and the potential of combination targeting of these pathways in the development of more effective anti-cancer therapies is reviewed by Vališ and Novák [4].

The impact of MAPK targeting on the immune response has been discussed in another review article focused on the current status of ongoing clinical trials investigating MAPK pathway inhibitors, such as BRAF and MAPK/ERK kinase (MEK) inhibitors, in combination with checkpoint inhibitors targeting programmed death protein 1 (PD-1), programmed death-ligand 1 (PD-L1), and cytotoxic T cell associated antigen-4 (CTLA-4) [5]. On the other hand, a research article showed that iron chelators induced apoptosis in osteosarcoma by activating the ROS-related MAPK signaling pathway, pointing to the possibility of exploiting iron chelators as a novel therapeutic strategy for osteosarcoma [6].

In the review from Donohoe and colleagues, the authors explored the links between MAPK signaling and obesity-related cancer, and found that several lines of evidence indicate that p38 and JNK are the MAPK involved in this scenario, whereas the role of ERK1/2 remains unclear [7]. On the other hand, the current knowledge in MAPK pathway alterations leading to gastric cancer, including the impact of *H. Pylori* on MAPK-triggering that may support derailing from gastric normal epithelium to GC, has been addressed in the review from Magnelli and colleagues [8]. 

The role of ERK5 in cancer onset and progression is well established, and MEK5/ERK5 signaling molecules are emerging as additional possible targets for cancer therapy [9]. Additionally, ERK5 activation has been described among the resistance mechanisms to RAS/RAF/MEK1/2-ERK1/2 targeting [10]. In the review article from Tubita et al., the authors provided an updated review on the mechanisms known to regulate ERK5 nuclear translocation, which is a key event to sustain cancer cell proliferation [11]. In the same review, the authors proposed ERK5 cyto-to-nuclear shuttling as an additional possible target for cancer therapy. Along this line, new mechanisms regulating ERK5 cyto-to-nuclear shuttling have been described in two research articles of this Special Issue. In the work by Pearson and colleagues, the authors describe a new intriguing mechanism involved in the activation of ERK5 and in its nuclear translocation [12], whereas the results provided by Erazo and colleagues point to a relevant role of ERK5 SUMOylation in its nuclear trafficking [13]. Finally, a cross-talk between the Hedgehog-GLI and MEK5-ERK5 pathways has been identified in melanoma cells in the article by Tusa et al., in which the authors identified a new HH-GLI/MEK5/ERK5 axis, the combined inhibition of which synergistically reduces melanoma cell proliferation [14].

The review article from Martínez-Limón et al. highlights the importance of elucidating critical aspects of p38 biology, such as isoform-specific functions or its apparent dual nature during tumorigenesis, thus opening up new possibilities for therapy with unexpected potential [15]. Along this line, another review focused on the recent insights into the exact role of the p38 MAPK pathway in response to currently available therapies for colorectal carcinoma, depicting an intricate scenario in which the p38 MAPK node presents many opportunities, as well as many challenges, for its exploitation for clinical purposes [16]. On the other hand, the possibility to target MK2, a direct target of p38 for the treatment of metastatic melanoma, is discussed in the review article by Petzelbauer [17].
